# Intrathymic somatotropic circuitry: consequences upon thymus involution

**DOI:** 10.3389/fimmu.2023.1108630

**Published:** 2023-06-22

**Authors:** Maria Danielma dos Santos Reis, Luciana Peixoto Veneziani, Felipe Lima Porto, Marvin Paulo Lins, Daniella Arêas Mendes-da-Cruz, Wilson Savino

**Affiliations:** ^1^ Laboratory of Cell Biology, Institute of Biological and Health Sciences, Federal University of Alagoas, Maceió, Brazil; ^2^ Oswaldo Cruz Institute, Oswaldo Cruz Foundation, Brazilian National Institute of Science and Technology on Neuroimmunomodulation (INCT-NIM), Rio de Janeiro, Brazil; ^3^ Laboratory on Thymus Research, Oswaldo Cruz Institute, Oswaldo Cruz Foundation, Rio de Janeiro, Brazil; ^4^ Rio de Janeiro Research Network on Neuroinflammation, Oswaldo Cruz Institute, Oswaldo Cruz Foundation, Rio de Janeiro, Brazil; ^5^ INOVA-IOC Network on Neuroimmunomodulation, Oswaldo Cruz Institute (IOC), Oswaldo Cruz Foundation, Rio de Janeiro, Brazil

**Keywords:** growth hormone, thymus, insulin-like growth factor-1, ghrelin, somatostatin, thymus involution, thymocyte development, thymic epithelial cells

## Abstract

Growth hormone (GH) is a classic pituitary-derived hormone crucial to body growth and metabolism. In the pituitary gland, GH production is stimulated by GH-releasing hormone and inhibited by somatostatin. GH secretion can also be induced by other peptides, such as ghrelin, which interacts with receptors present in somatotropic cells. It is well established that GH acts directly on target cells or indirectly by stimulating the production of insulin-like growth factors (IGFs), particularly IGF-1. Notably, such somatotropic circuitry is also involved in the development and function of immune cells and organs, including the thymus. Interestingly, GH, IGF-1, ghrelin, and somatostatin are expressed in the thymus in the lymphoid and microenvironmental compartments, where they stimulate the secretion of soluble factors and extracellular matrix molecules involved in the general process of intrathymic T-cell development. Clinical trials in which GH was used to treat immunocompromised patients successfully recovered thymic function. Additionally, there is evidence that the reduction in the function of the somatotropic axis is associated with age-related thymus atrophy. Treatment with GH, IGF-1 or ghrelin can restore thymopoiesis of old animals, thus in keeping with a clinical study showing that treatment with GH, associated with metformin and dehydroepiandrosterone, could induce thymus regeneration in healthy aged individuals. In conclusion, the molecules of the somatotrophic axis can be envisioned as potential therapeutic targets for thymus regeneration in age-related or pathological thymus involution.

## General features on the role of the GH axis upon the immune system

The nervous, immune, and endocrine systems act together through a multidirectional communication mechanism involving hormones, neurotransmitters, cytokines, and anatomical structures, contributing to maintaining homeostasis. Among the substances that participate in these interactions stand out the molecules of the somatotropic axis, including growth hormone (GH, somatotropic hormone or somatotropin), somatostatin, insulin-like growth factors (IGFs) and ghrelin. Aside from their classical effects on the body’s metabolism, these peptides have been studied as immunomodulators, acting in the development, activity and function of organs and cells of the immune system.

GH is a 22 kDa polypeptide secreted by somatotrophs in the pituitary gland in a pulsatile manner in response to the GH-releasing hormone (GHRH) produced by neurons in the hypothalamus (1). GH acts directly or indirectly on the target cells stimulating the production of insulin-like growth factors (IGFs), especially IGF-1. GH also influences the metabolism of carbohydrates, lipids, and minerals. In this context, two important actions can be related to GH: an action like that of insulin characterized by hypoglycemia, increased protein synthesis, glycogenesis, and lipogenesis; and another late action, in which the opposite occurs, with hyperglycemia, hyperinsulinemia, increased lipolysis, and reduced glucose metabolism, corresponding to the primary physiological effects related to GH (2).

Somatostatin, formerly called a somatotropin-release inhibiting factor, is a peptide hormone found in two biologically active forms with 14 or 28 peptides named somatostatin-14 and somatostatin-28, respectively (1, 3). This hormone can be produced by hypothalamic neurons and elicit their inhibitory action by binding to G-protein-coupled somatostatin receptor (SSTRs) (1, 3). It is widely expressed in different tissues, exerting inhibitory effects on the release of hormones such as GH, thyroid-stimulating hormone (TSH), insulin and glucagon, and acting in the immune response (3, 4). Because of its inhibiting effects upon GH, somatostatin receptor agonists are also used to treat acromegaly (5).

The pulsatile mode of GH release makes its secretion control more complex than the dichotomy GHRH and somatostatin (6). GH secretion can also be stimulated by other peptides called GH secretagogues (GHS), including thyrotropin-releasing hormone (TRH), pituitary adenylate cyclase-activating polypeptide (PACAP) and ghrelin (7–9). In humans, PACAP and TRH appear to play only a supporting role in controlling the somatotropic axis, whereas GHRH is essential for GH release (9). Ghrelin is a peptidic hormone produced mainly by X/A-like enteroendocrine cells in the stomach in response to negative energy balance, inducing the orexigenic response and GH secretion by the pituitary gland through the growth hormone secretagogue receptor (GHS-R) (10). It can be found across tissues including the pituitary and hypothalamus, suggesting possible direct and indirect action on GHS-R expressing somatotrophs, stimulating GHRH and GH (1, 11).

Many GH actions are mediated indirectly by the IGFs such as IGF-1. This growth factor is a member of a group of polypeptides structurally related to insulin, composed of 70 amino acids organized into α and β chains linked by disulphide bonds (12). The role of IGF-1 is regulated by insulin-like growth factor binding proteins (IGFBPs), which act as carriers of IGFs in the plasma, guiding them to target cells (13). Several cell types express IGF-1 and its receptor, IGF-1R, allowing autocrine and paracrine modes of action (14).

In addition to the classic pathways, the somatotropic circuit is involved in the development and function of immune cells and organs. Receptors for GH, IGFs, ghrelin and somatostatin are expressed in B and T lymphocytes, natural killer cells, monocytes, neutrophils, macrophages, and in the bone marrow, thymus, spleen, and lymph nodes (8, 15–17). Moreover, cells of the immune system, including the microenvironmental cells in lymphoid organs, can produce these peptides, potentially establishing autocrine and/or paracrine mechanisms of action, in addition to their systemic effects (8, 18–20), acting in the development of myeloid and lymphoid cells, during physiological and pathological conditions (21–24). They can also modulate the function of immune cells in terms of proliferation, cytokine production, antigen response and antibody production (8, 25–28).

Notably, the influence of the somatotropic circuitry on the physiology of the immune system also seems essential in the host’s defense against pathogens. For instance, somatostatin produced by gastric D cells under IL-4 stimulation is required to control *Helicobacter*-induced gastritis, reducing inflammation and bacterial proliferation (29). In the same vein, ghrelin can reduce lipopolysaccharide (LPS)-induced endotoxemia through the inhibition of proinflammatory cytokines (30). In hypophysectomized animals, GH treatment induced macrophage bactericidal activity against *Salmonella typhimurium*, thus favoring the survival of the infected animals (31). Furthermore, in a mouse model of *Escherichia coli*-induced sepsis, an increase in defense against infection was observed after short-term treatment with both GH and IGF-1 (32). Moreover, GH-induced protection was demonstrated for *Mycobacterium avium*, herpes simplex virus type 1 and *Trypanosoma cruzi* infections (33–35).

A particular target organ for the molecules of the somatotropic axis is the thymus, a primary lymphoid organ responsible for the maturation of immunocompetent T lymphocytes. Since the 1960^th^ decade, many studies with genetically GH-deficient or hypophysectomized animals as well as with transgenic mice hyperexpressing GH, IGF-1 or IGF-2, have shown that these hormones are relevant for thymus physiology (36–38), and so is the production of ghrelin in the maintenance of the thymus throughout life (39, 40). Of note, somatostatin and its receptors are also expressed in the thymus, thus providing the molecular basis for an intrathymic control of the somatotropic axis independently of the pituitary gland (41). Accordingly, the involvement of the somatotropic axis is highlighted during the process of thymus involution, in which significant alterations of the thymic functions are observed, with reduced production of GH, IGF and ghrelin, somehow mimicking the reduction of the systemic levels of these hormones in the elderly (42, 43).

Although most preclinical data indicate the relevance of this circuitry in the immune system, particularly in the thymus, clinical studies on the effect of these molecules upon the human immune function show distinct results. In individuals with isolated congenital GH deficiency (IGHD) due to a mutation in the gene of the GHRH receptor, no clinically relevant immune alterations were found, despite a small spleen, low IgG levels and more incidence of periodontal infections than age-matched controls (44, 45). Similar observations were described using the *Ghrh* KO mice, with a reduction in the size of the spleen together with the diminishment of the B lymphocyte numbers (46). Moreover, these animals were more susceptible to *Streptococcus pneumoniae* infection (47). Like IGHD individuals, patients with Laron syndrome (IGF-1 deficiency induced by GH insensitivity) have normal immune functions. However, they are more prone to develop severe lung infections that lead to death (48).

Differently, severe immunodeficiency is observed in another form of GH insensitivity due to a mutation in the signal transducer and activator of transcription 5B (STAT5B) gene, with reduced IGF-1 production, impaired response to infections and high production of prolactin (PRL) (49). Secondly, in GH-deficient HIV-positive children treated with highly active antiretroviral therapy (HAART), fewer circulating CD4+ lymphocytes, smaller thymus, and decreased naive T cells were observed (50). Since HAART helps the recovery of thymus function during HIV infection (51), the persistence of the immunosuppression can be linked with the impairment of the GH axis. Interestingly, patients with the coronavirus disease 2019 (COVID-19) also showed low levels of GH and IGF-1 in the bloodstream, associated with lung inflammation, reinforcing the possible involvement of the somatotropic axis in triggering and/or maintaining an appropriate immune response (52).

Overall, the molecules of the somatotropic axis can be considered as potential therapeutic alternatives when an improvement in the immune system is desired, especially in the recovery of the thymus during acute and chronic injuries that result in thymus involution, as detailed below in the next sections.

## The thymic microenvironment and T-cell differentiation

The development of functional T lymphocytes occurs in the thymus, a primary lymphoid organ in vertebrates. In mammals, the thymus is a mediastinal organ, with the parenchymal tissue partially divided into lobules; each one having cortical and medullary regions. The cortex, densely stained by haematoxylin-eosin, is packed with large amounts of immature thymocytes intermingled with sparse non-lymphoid microenvironmental cells. The medulla contains more mature thymocytes, in addition to prominent microenvironmental cells. In the medulla, there is an accumulation of phenotypically mature T cells, that have passed the positive and negative selection processes and are able to egress the thymus and populate peripheral lymphoid organs (19).

The thymic microenvironment is unique because of its three-dimensional architecture, in which interactions between developing thymocytes and stromal cells take place. There are several cell types composing the thymic microenvironment including thymic epithelial cells (TECs) (quite heterogeneous in their subsets), fibroblasts, mesenchymal cells, neural and vascular cells, as well as hematopoietic-derived dendritic cells, macrophages, and B lymphocytes (53). Thymic microenvironmental cells are responsible for producing most of the inductive signals for thymocyte differentiation, including cytokines, chemokines, thymic hormones, other soluble factors, as well as cell-cell interactions like those mediated by the T-cell receptor (TCR) and class I or class II molecules of the major histocompatibility complex (MHC). Moreover, ephrins, integrins, and extracellular matrix molecules, as well as Notch1 receptors present on thymocytes and the Notch ligands Delta-like (DLL)-1 and DLL4 present on TEC, are among the cell-cell interactions that mediate thymocyte differentiation and contribute to the general architecture of the thymic microenvironment (19, 54, 55).

Thymic epithelial cells are the most prominent cellular component in the thymic microenvironment, producing the necessary chemotactic stimuli for the entry of progenitor cells into the thymus. They also interact with developing thymocytes, inducing their proliferation and positive and negative selection, ultimately guiding the intrathymic CD4^+^ and CD8^+^ T-cell differentiation. TECs are found in the cortex (cortical TECs; cTECs) and the medulla (medullary TECs; mTECs) of the thymic lobules. Early stages of thymocyte differentiation are directed by cTECs, including positive selection (53). In contrast, mTECs, which are morphologically distinct from cTECs, play an essential role in thymocyte negative selection, being crucial for the development of central immunological tolerance (53).

In recent years, technological advances have improved studies concerning the human thymus. A combination of single-cell techniques and functional assays have been applied to identify subtypes of cTECs and mTECs, which were identified using single-cell RNA-sequencing (scRNA-seq) analysis (56). Human TECs were characterized based on a combination of known TEC markers and a list of differentially expressed genes in three superclusters, defined by the expression of epithelial cell adhesion molecule (*EPCAM*) and keratin 8 (*KRT8*) genes, and nine sub-clusters, defined by the expression of forkhead box N1 (*FOXN1*), proteasome subunit beta 11 (*PSMB11*), lymphocyte antigen 75 (*LY75*), claudin 4 (*CLDN4*), and autoimmune regulator (*AIRE*) genes (57).

Although TECs are the most studied thymic microenvironmental cells, numerous studies have described the participation of other thymic cell types in thymocyte development, and in the homeostasis of the thymic stroma itself. This includes, among others, dendritic cells (DC), B lymphocytes, fibroblasts, and endothelial cells. For example, thymic endothelial cells and thymic fibroblasts can produce stem cell factor (SCF), and thymic endothelial cells also express DLL4. Both molecules are crucial for the homing and commitment of T-cell precursors to the T-cell lineage (58, 59). Furthermore, thymic mesenchymal cells can modulate thymocyte viability and differentiation (60), highlighting the importance of microenvironmental cells other than TECs for thymus function.

As briefly mentioned above, the interactions of developing thymocytes with microenvironmental cells occur through cell-cell contact and *via* soluble factors and cell-extracellular matrix (ECM) contact. The ECM in the thymus is composed of a large variety of molecules, including among others, fibronectins, laminins, and collagens, also acting as a reservoir for soluble factors (61, 62). Additionally, integrin-type receptors for these ligands, which are expressed by both developing thymocytes and microenvironmental cells, comprise very late antigen (VLA)-4 (α4β1, CD49d/CD29), VLA-5 (α5β1, CD49e/CD29) and VLA-6 (α6β1, CD49f/CD29) (63, 64).

The process of thymocyte differentiation is complex including events of cell differentiation, migration, proliferation, and death. Along with differentiation, developing thymocytes migrate along cortical and medullary regions of the thymic lobules, receiving essential signals from the microenvironmental cells, particularly the thymic epithelium (65). The continued homing of bone marrow progenitors into the thymus is necessary to maintain T lymphopoiesis since thymic progenitors lose their self-renewal potential. Common lymphoid progenitors (CLP) or their immediate cellular progeny enter the thymus in the corticomedullary junction, where they interact with the vascular endothelium *via* P-selectin.

Within the thymic tissue, cTECs deliver interleukin (IL)-7, SCF, and express the canonical DLL4, which are indispensable for survival, differentiation, and early T-lineage commitment (66). Developing thymocytes initially do not express CD4 or CD8 coreceptors (bearing the double-negative – DN – phenotype), being further subdivided into four stages (DN1 to DN4), according to the expression of c-Kit, CD25, and CD44 markers in mice. DN1 thymocytes (c-Kit^+^CD44^+^CD25^−^) are seen in the cortex, close to the corticomedullary junction. DN2 thymocytes (c-Kit^+^CD44^+^CD25^+^) migrate to the middle of the thymic cortex while rearranging the TCRβ, TCRγ, and TCRδ gene loci, which begin in DN2 cells and are completed in DN3 cells. These rearrangements occur through V(D)J recombination, which allows the generation of a high diversity of antigen receptors. Then, the DN3 (c-Kit^−^CD44^−^CD25^+^) stage represents the checkpoint for αβ or γδ lineage differentiation. The γδ -selected cells will express the membrane γδ TCR, becoming the so-called γδ T lymphocytes. Some γδ T cells emigrate from the thymus while still immature, but others complete their maturation within the organ before migrating to the periphery. Mature γδ T cells possess a defined effector profile (associated with interferon-gamma (IFN-γ) or IL-17 production) and can play several roles in the immune system like as protection against tumors and infections (67, 68).

TCRβ-selected cells proliferate in the subcapsular cortex and differentiate into DN4 (c-Kit^−^CD44^−^CD25^−^) to rapidly become double-positive (DP) thymocytes (CD4^+^CD8^+^) (69). Cells reaching the DP stage then begin rearranging the TCR α chain gene. The efficient rearrangement leads to the expression of the TCR αβ complex on thymocyte membranes. These receptors are functionally tested for the recognition of self-antigen loaded-MHC molecules (positive selection). Thymocytes that express a TCR with low affinity for the self-peptide are induced to differentiate into naïve T cells. However, thymocytes that express a TCR with high affinity to MHC are eliminated by apoptosis. Beyond that, some of these autoreactive cells differentiate into regulatory T lymphocytes (Tregs). If the TCR is unable to bind to the MHC, thymocytes “die by neglect” – also by apoptosis (70).

Negative selection involves the clonal deletion of autoreactive T cells, preventing their escape into peripheral tissues. This process results in the apoptosis of those thymocytes bearing TCRs with a high affinity for a given self-peptide. In addition, some CD4SP thymocytes start to express the transcription factor Foxp3, resulting in the formation of Tregs cells, which leave the thymus and populate the peripheral tissues, limiting functional responses of the few autoreactive T cells that escaped negative selection (71).

The thymic medulla is critical for the induction of central tolerance in developing T lymphocytes (72). mTECs express numerous tissue-specific autoantigens (TSA –normally found only in specific peripheral tissues) in association with MHC molecules. Several TSA genes are controlled by nuclear factors as AIRE and the FEZ Family Zinc Finger 2 (73). The medulla is also the region where DCs and B cells are frequently found, contributing to the induction of self-tolerance (74). Recently, it has been described that plasmacytoid DC can migrate to the thymus to present peptides derived from the microbiome and modulate the developing thymic cells (75). Thus, future studies on the thymus-gut axis may provide more light in the balance between healthy immunity and autoimmune diseases.

Once mature, naïve lymphocytes exit the thymus following a sphingosine-1 phosphate gradient around blood vessels at the corticomedullary junction. These cells cross the basement membrane of the vessels, entering the perivascular space between the endothelial cells and the pericytes. The last step is reverse trans endothelial migration, in which they leave the perivascular space and enter the bloodstream, joining the pool of peripheral T cells as recent thymic emigrants (RTE) (76).

The physiology of the thymus can be disturbed by the influence of external and internal factors, leading, for example, to thymic atrophy or thymic involution. Irradiation, pregnancy, infections, immunosuppressant status, and undernutrition are known to trigger acute thymus involution, characterized by increased death of DP thymocytes and intrathymic inflammation (77–82). Also, a physiological long-term thymic atrophy is observed during aging (chronic thymus atrophy), with remarkable changes in the microarchitecture and composition of the thymic microenvironment, such as an increase in adipose cells and a decrease and disorganization of cortical and medullary regions, together with downregulation of TEC-related transcriptional factors (83–86). In this physiological condition, there are quantitative and qualitative degenerations of TECs, in parallel with the increase of adipocytes in the organ, leading to a less efficient T cell selection, decreased self-antigen presentation, and decreased output of naïve T cells with limited TCR diversity (87, 88). This limits the adaptive immune response against new pathogens, even though, in humans, the number of naïve T lymphocytes remains close to the numbers found in young individuals through the proliferation of these cells in peripheral lymphoid structures (85, 89, 90). The impact of senescence on other thymic cells and their role in thymus involution are the subject of further studies.

Despite changes in thymus function and structure, an endogenous regenerative program is observed after acute injuries, mainly mediated by the expression of IL-7, IL-22, IL-23, bone morphogenetic protein-4 (BMP-4), keratinocyte growth factor (KGF), and Receptor activator of nuclear factor kappa-Β ligand (RANKL), which induce TEC survival and proliferation, along with thymocyte maturation (79). Similar effects are seen in thymus repair following sex steroid inhibition, where the reconstitution of thymic tissue seems to be mediated by enhancing hematopoietic potential and replenishing TEC subtypes through differentiation of TEC progenitors (91, 92).

## Expression of GH, IGFs, ghrelin, somatostatin, and corresponding receptors in the thymus

The existence of an intrathymic somatotropic circuitry has been suggested since GH, IGFs, ghrelin and somatostatin have been detected in the thymus (19, 41). In addition to the classical endocrine pathway, these molecules can act *via* paracrine and/or autocrine signaling, influencing the physiology of both microenvironmental and lymphoid compartments. Interestingly, this thymic hormonal network may also influence pituitary gland hormonal release since a conditioned medium from rat thymic microenvironmental cells can induce the secretion of GH, PRL, and luteinizing hormone by cultured anterior pituitary cells (93). This thymus-neuroendocrine crosstalk is reinforced by the presence of specific functional receptors for GHRH in rat thymocytes able to enhance GH-specific mRNA after GHRH stimulation (94, 95).

GH production in the thymus can also be induced by the ghrelin secreted by both TEC and thymocytes (20, 30). This hormone mediates GH release by acting upon the G-protein-coupled receptor GHS-R (19, 96). The receptor was detected in the thymic medulla and across thymocyte populations, with the highest expression in the DP thymocytes (39, 40).

Intrathymic GH may be subjected to inhibition similarly to that observed in the pituitary gland, as thymic cells can produce somatostatin and express the corresponding receptors (16, 41, 97, 98). Production of somatostatin was shown in the thymic medulla, and mRNA-positive cells for the protein and its receptors (SSTR1, SSTR2A, and SSTR3) were detected in the human thymic tissue (16, 99). More specifically, mRNAs for somatostatin and SSTR1 and SSTR2A receptors were detected in TEC (16). Differently, mRNAs for SSTR2A and SSTR3 were seen on thymocytes, although a variation in the number of copies was observed in the immature (CD2^+^CD3) and intermediate/mature (CD3^+^) phenotypes, with the most immature expressing a higher number of SSTR2A copies. Intermediate/mature thymocytes exhibited a higher number of mRNA copies for SSTR3 (100).

Whilst mRNA for somatostatin was only identified on human TECs, mRNA for cortistatin, a peptide with structural similarity to somatostatin able to bind to somatostatin receptors subtypes, was identified in TECs and human thymocytes, with higher expression in TECs (15, 16). Further evidence showed that mRNA cortistatin levels are higher in TECs as compared with thymocytes (15).

In the thymus, the GH is produced by TEC and thymocytes, and TEC can produce IGF-1 under GH stimulation (101, 102). The GH receptor, GHR, was detected in the human thymus samples, in human TEC cell line and in all thymocyte subsets, with the highest percentage of positive cells seen in immature subtypes (101, 103). GHR belongs to the class I cytokine receptor family and upon binding it triggers the Janus kinase (JAK)-2/Signal transducer and activator of transcription (STAT)-5 pathway activation and synthesis of IGF-1 (104). Therefore, the GH-induced responses in thymic cells can be directly mediated by the binding to GHR or indirectly by the synthesis of IGF-1. Indeed, there was shown that GH effects on TEC can be inhibited by anti-IGF-1 and anti-IGF-1R antibodies (102, 105). Studies in BALB/c mice showed that the GH, IGF-1, and IGF-2 genes are expressed in the thymus since early embryonic stages and decrease after birth, indicating the role of the somatotropic axis in thymus development (106).

IGF-2 seems to be the predominant insulin-related peptide expressed by human and rat TECs, as it displayed a higher concentration in the human thymus as compared to IGF-1 (107–109). Human and rat thymus showed strong immunoreactivity for IGF-2, mainly in epithelial cells of the subcapsular cortex and in the medulla (108).

The intrathymic expression of molecules comprised in the somatotropic axis is summarized in **Table 1**.

**Table 1:** Expression of somatotropic ligands and corresponding receptors by thymic cells.

Molecule	Cell type expressing somatotropic ligands		Cell type expressing the corresponding receptors		References
	TECs	Thymocytes	TECs	Thymocytes	
GH	+	+	+	+	(101)
IGF-1	+	+	+	+	(110)
IGF-2	+	+	+	+	(107, 108, 111)
GHRH	ND	+	ND	+	(94, 95)
Ghrelin	+	+	+	+	(30, 39, 40)
Somatostatin	+	+	+	+	(23, 98, 100)

GH, Growth hormone; IGF, Insulin-like factor; GHRH, Growth hormone-releasing hormone; TEC, Thymic Epithelial Cells; ND, Not Determined.

## Pleiotropic effects of GH, IGF-1, ghrelin, and somatostatin upon the thymus: lessons from genetically engineered mice and human clinical settings

The first observations about the influence of GH on the thymus date back to the 1960s, with studies in *Snell-Bagg* mice. These animals exhibit abnormal anterior pituitary development and lack somatotropic cells, reaching 1/3 normal size when adults. GH therapy in these animals reversed thymic atrophy, restored microarchitecture and cellularity in the organ cortex, and recovered thymic DNA synthesis (36). Currently, it is widely known that GH has several pleiotropic effects on the thymus, in both lymphoid and microenvironmental compartments. TECs produce GH but also express the GH receptor and proliferate more in the presence of this hormone *via* the upregulation of cyclin A and cyclin-dependent kinases (112). GH modulates several functions in the thymic microenvironment, including increased secretion of cytokines (IL-1α, IL-1β and IL-6), the chemokine CXCL12, and the thymic hormone thymulin (113).

Most likely, at least part of the GH effects upon the thymus is related to the modulation of cell adhesion and migration. Accordingly, *in vitro* treatment of murine and human TECs with GH resulted in increased deposition of fibronectin and laminin and their respective receptors, VLA-5, and VLA-6 (114, 115). Also, thymocyte adhesion to GH-treated TECs is upregulated, and exposure of these cells to anti-GH antibodies or anti-ECM abolished the effect (116). Moreover, GH increased the adhesive capacity of thymocytes to fibronectin, Intercellular Adhesion Molecule 1 (ICAM-1) and Vascular Cell Adhesion Protein 1 (VCAM-1), as well as on thymic endothelial cells (112, 117, 118). Responsiveness of the thymic endothelium to GH can also be seen by the mitogenic effect of GH upon these cells, together with changes in their morphology and increased deposition of fibronectin and laminin (119). In the same *in vitro* model, GH stimulated cell fugetaxis – as ascertained in transwell chamber migration assay and increased the formation of capillary-like structures in Matrigel^®^-coated plates. Further *in vitro* studies revealed an improvement in the deposition of fibronectin by these endothelial cells when co-cultured with GH-pretreated thymocytes. In a transendothelial cell migration assay, large numbers of GH-treated thymocytes, mainly the mature CD4^-^CD8^+^ subset, migrated through the thymic endothelium following IGF-1 stimulation (117).

In transgenic mice for the bovine GH, with high circulating levels of the hormone (120), an increase in the intrathymic contents of cell migration-related moieties such as laminin and CXCL12 was found. Additionally, *ex-vivo* migration of thymocytes towards laminin and CXCL12 molecules was potentiated by GH, both in GH transgenic animals and in intrathymically GH-injected mice, as well as in human thymocytes exposed to GH (38, 115). Other *in vivo* experiments demonstrated that GH favors the release of RTEs, mainly CD4^+^CD8^-^, to the subcutaneous and mesenteric lymph nodes, but not to the spleen, inducing a differential distribution of RTEs in peripheral lymphoid organs. It was also observed that the expression of L-selectin (CD62L) was increased in these cells (121). Similar data were reported regarding human RTEs. HIV-positive patients who received GH as an adjuvant treatment during their antiretroviral therapy showed an increase in the thymic export of CD4^+^ T lymphocytes (122). These preclinical and clinical data show that GHs affect the migratory patterns of thymocytes, including the export of mature T cells to the periphery.

At least some of the GH effects on the thymic microenvironment are mediated by IGF-1. Murine and human TECs express the IGF-1 receptor and produce IGF-1 in response to GH stimulation (110). This factor also influences the production of thymic hormones and extracellular matrix molecules by TECs and stimulates a mitogenic response and the production of IL-6 by thymocytes (117). CXCL12 and Chemokine (C-C motif) ligand 25 (CCL25) expression was increased in both cTECs and mTECs after IGF-1 administration (123). IGF-1 also favors adhesion and acts as a chemoattractant, inducing thymocyte migration (116, 117). Also, as mentioned above, the GH effects on cultured human TEC can be prevented by anti-IGF-1 and anti-IGF-1 receptor antibodies (116).

IGF-2 is also produced in the thymus and plays a role in the organ. Interestingly, in transgenic mice overexpressing the human transgene encoding IGF-2, body and organ weights were similar to controls, except for the thymus, which showed a significant increase in both growth and weight, suggesting a paracrine/autocrine action (37). In these animals, thymic cellularity was increased in all thymocyte subsets, and the rise in the number of CD4^+^CD8^-^ thymocytes contributed to the presence of more CD4^+^ T cells in the spleen (124). In a second vein, it has been shown that in murine fetal thymic organ cultures (FTOCs) treated with anti-IGF-2 antibodies, T cell differentiation was severely inhibited at early stages (DN thymocytes), strongly indicating the participation of IGF-2 in thymopoiesis (111). Furthermore, the thymic microenvironment of IGF-2 transgenic mice is pleiotropically affected, with abnormal thymic epithelial cell network, higher circulating levels of thymulin and increased fibronectin and laminin contents, both *in situ* and in TEC cultures (125). Importantly, thymocytes derived from IGF-2 transgenic mice had increased *ex vivo* migratory activity, with a higher frequency of DN and DP thymocytes.

IGF-2 is also involved in T-cell negative selection since the self-antigen presentation of IGF-2 epitopes might play a role in the induction of central tolerance toward the insulin family of peptides (126). This potential role becomes even more intricate since some viral infections can reduce or increase intrathymic IGF-2 expression, such as infection with coxsackievirus B4 and herpes simplex virus 1, respectively (127). Future studies will hopefully throw more light on this issue.

Ghrelin, a GH secretagogue, is also relevant in thymus physiology. Gene ablation of ghrelin and its receptor leads to the loss of TEC and an increase in adipogenic fibroblasts in the thymus suggesting a possible role in the transition of TEC to a mesenchymal phenotype (39). In addition, the authors showed that the compromised thymic microenvironment due to the lack of ghrelin signalling was associated with a reduced number of naïve T cells (39).

As mentioned above, somatostatin is another member of the intrathymic GH-related biological circuitry. In the human thymus, somatostatin plays an inhibitory role as the treatment of isolated human TECs and thymocytes with different concentrations of somatostatin led to a decrease in proliferation, as seen by thymidine incorporation (16, 100). Furthermore, octreotide, a somatostatin analog that binds to SSTR2 with high affinity, was able to decrease TEC proliferation, although its effect on thymocyte proliferation was only seen after the isolation of immature thymocytes (CD3^-^negative) that mainly expressed SSTR2 (100). Yet, data obtained from mouse FTOC cultures indicate an alternative hypothesis, as somatostatin treatment led to an increase in the progression of the most immature DN to DP phenotype with increased cellularity (24). Furthermore, somatostatin enhanced thymocyte migratory response, suggesting that it could also act as a chemoattractant to these cells.

**Figure 1:**
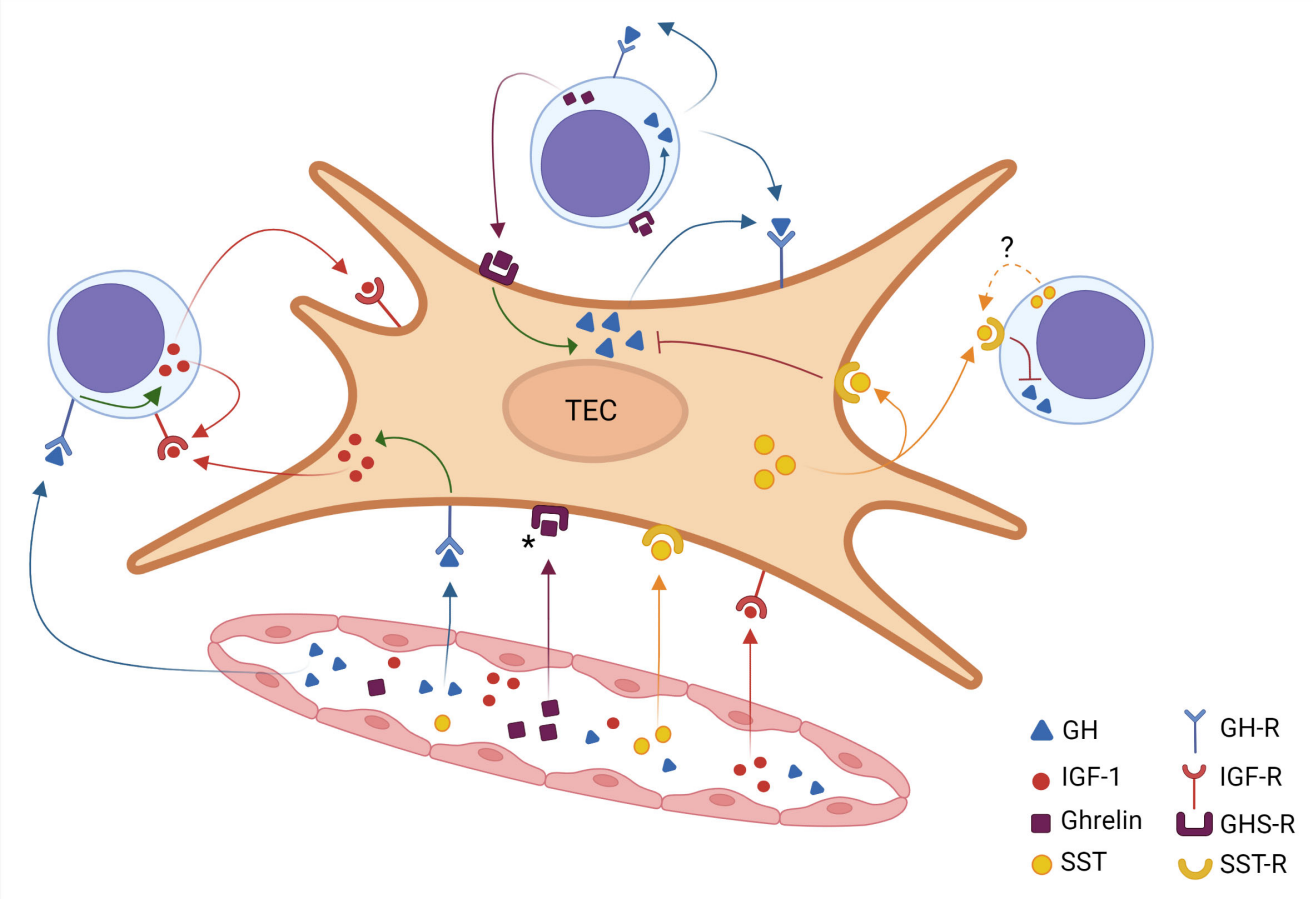
Intrathymic somatotropic axis network. A thymic epithelial cell (TEC) is shown in the center of the figure, surrounded by three thymocytes and a blood vessel below. The somatotropic molecules reach the thymus through the bloodstream, thus characterizing an endocrine pathway. These hormones (identified by color) diffuse from the inside of the blood vessel into the organ and bind to their receptors on TEC and thymocytes. The ghrelin receptor signaling induces the intracellular production of GH, which in turn promotes the IGF-1 production. On the other hand, somatostatin inhibits the GH production. The figure also reveals the various possibilities of paracrine and autocrine action for each hormone, which is identified by the corresponding color. Filled colored arrows indicate well-established evidence for these hormone interactions, the dotted arrow in the somatostatin pathway indicates that the corresponding scientific evidence needs further studies. Asterisk in the GHS-R indicate that expression of this receptor has not yet been demonstrated in all TECs. Created with BioRender.com.

The data summarized above show that a somatotropic circuitry can modify the interactions between developing thymocytes with TECs, also influencing the exit of mature thymocytes through the thymic endothelium. Although further studies are needed, the existing data led us to conceive a general scheme (depicted in **Figure 1**) describing the rather complex somatotropic endocrine/paracrine/autocrine circuitry in the thymus.

## Thymus involution can be reverted by the activation/restoration of the GH axis

It is well known that the thymus can be affected by extrinsic and intrinsic stress factors, leading to a reduction in size and a decline in the production of immunocompetent T lymphocytes in a process called thymus involution. Importantly, this process has been linked with the impaired secretion of GH, IGF-1, and ghrelin, both systemically and intrathymically, raising the hypothesis that thymus atrophy is associated with an impaired intrathymic somatotropic circuitry (42, 128–131).

During the response to stressors, such as an acute infection, there are changes in neuroendocrine circuits, with activation of the hypothalamus-pituitary-adrenal axis (HPA) to release glucocorticoids (GC) in conjunction with an increase in catabolic pathways and a decrease in anabolic factors as GH and IGF-1. It has been shown that the infection with the lymphocytic choriomeningitis virus decreased GH production in rat pituitary cells without any structural changes (128). Similarly, the Zika virus infection in newborn mice caused a reduction in the production of GH due to hypothalamic damage (129). The central control of GH secretion is also impaired in Chagas disease since *Trypanosoma cruzi*-infected pituitary cells have decreased production of not only GH but also PRL (132, 133). Of note, rodents with endotoxemia also exhibited reduced contents of serum GH and IGF-1 (134).

The impact of these systemic changes upon thymus atrophy is not completely known. However, some clinical data from patients with GH deficiency (GHD) may shed light on this matter. In a study with HIV-positive children after HAART, the ones with GHD had fewer circulating CD4+ lymphocytes, smaller thymus, and decreased number of naïve T cells (50). It was also shown that these patients had decreased IGF-1 as well as IL-7 serum levels (50). It is known that HIV infection impairs thymus function and reduces the generation of T lymphocytes and that HAART helps the recovery of the thymus function (50, 51). That GHD children had impaired thymus function after four years of HAART, indicating that a functional somatotropic axis may be required for the therapy to succeed (50). Corroborating these observations, clinical trials with HIV-positive adults demonstrated that GH treatment augmented thymus size and the number of naïve T CD4+ lymphocytes in the blood and improved the specific T-cell response to the virus (26, 122). Pioneer work by Laura Napolitano and coworkers showed that, although the thymus of HIV patients was reduced after the interruption of the GH treatment, the beneficial effects on CD4^+^ naïve cells were long-lasting, up to 12 months after the discontinuation of the treatment (122). Yet, the number of patients evaluated in this study was too low to confirm these lasting effects, indicating that more clinical studies with larger cohorts are crucial to ultimately determine the actions and benefits of GH intervention in acute thymus involution.

The same benefits can likely be replicated in the case of other pathologies where the thymus is failing. In this regard, promising results have been obtained in preclinical *in vivo* and *in vitro* studies using GH as therapy for Chagas disease, where the hormone was able to reduce parasitemia together with an increase in the response against the protozoan and reduced infection of the myocardial tissue (35, 135). Interestingly, in the experimental Chagas disease model, there is an increase in the intrathymic and serum glucocorticoid contents, which are related to the death of DP thymocytes and the atrophy of the organ (81, 136). Yet, the benefits of GH therapy in *T. cruzi*-induced thymus atrophy remain to be better investigated.

Although the precise role of somatotropic axis molecules in acute thymus atrophy has yet to be determined, the effects of these peptides upon age-related thymic atrophy are the subject of various preclinical and clinical studies (summarized in **Table 2**). Accordingly, treatment of old mice with GH restored the proliferative capacity of the thymocytes and secretion of the thymic hormone thymulin (137). Also, when aged rats were treated with human or rat GH, the thymus architecture was restored, with increased numbers of thymocytes and well-defined cortical and medullary zones, similar to the patterns seen in the young control rats (138). Additionally, the administration of GH in 14-month-old BALB/c mice, not only increased thymic cellularity but also increased the number of early thymic progenitors as well as RTEs (42). GH also reduced lipotoxicity and pro-inflammatory profile in the aged thymus (139).

**Table 2:** Preclinical and clinical studies using GH, IGF-1, and ghrelin to reestablish thymic function.

Treatment regimen	Type of the study	Thymus involution condition/model	Species	Effects in the thymus	References
GH human recombinant, subcutaneous, 3-1.5 mg/d (30–40 μg/kg/day)	Clinical trial	HIV+ infection	Human	↑ Thymus size (transient) ↑ CD4+ cells in peripheral blood	(122)
GH human recombinant, subcutaneous, 4 mg/day, 12 weeks, alternate-day or twice-per-week dosing	Clinical trial	HIV+ infection	Human	↑ CD4+ cells in peripheral blood ↑ T-cell response to the virus	(26)
Ovine GH, subcutaneous, 2μg/g/day	Preclinical	Aging	C57BL/6 mice	↑ Thymocyte proliferation ↑ Thymulin secretion	(137)
human recombinant GH, subcutaneous, 1 mg/kg, twice daily	Preclinical	Aging	Wistar-Furth rats	Restored Thymic architecture ↑ Thymic cellularity	(138)
GH, Subcutaneous (osmotic pumps), for 14 days	Preclinical	Aging	BALB/c mice	↑ Thymic cellularity ↑ ETP ↑ RTE	(42)
Ovine GH, Subcutaneous (osmotic pumps), 40μg/day/mouse for 14 days	Preclinical	Aging	C57BL/6 mice	↓ Lipotoxicity ↓ Pro-inflammatory profile in the aged thymus	(139)
GH human recombinant 0.015 mg/kg + dehydroepiandrosterone (DHEA) 50 mg + metformin 500 mg for 4 weeks	Clinical trial	Aging	Human	↑ Thymus size ↑ RTE ↑ Naïve T lymphocytes ↓ Thymic fat content	(140)
IGF-1 Human recombinant, subcutaneous (mini-osmotic pumps), 100 μg/day for 14 days	Preclinical	Aging	BALB/c and C57BL/6 mice	↑ Thymic cellularity	(141)
IGF-1 human recombinant, subcutaneous (osmotic minipumps), 100 μg/day for 14 days	Preclinical	Immunosuppression	C57BL/6J and CBA/J mice	↑ Thymic cellularity (DN thymocytes)	(142)
Ghrelin, orally and daily, 5 mg/kg for 3 weeks	Preclinical	Aging	BALB/cJ and C57BL/6J mice	↑ Thymic weight and cellularity	(118)
Ghrelin, subcutaneous (mini-osmotic pumps), 1.25 μg/hour for 2 weeks	Preclinical	Aging	BALB/c, C57BL/6 and Ghrelin and GHS-R knockout mice	↑ Thymic size and cellularity ↑ cTEC and mTEC ↑ Thymocyte progenitors and naïve lymphocytes in the periphery	(143)
Ghrelin, intraperitoneally, 100 μg/kg, daily for 7 days	Preclinical	Stress	C57BL/6	↑ Thymic weight and cellularity ↓ Thymocyte apoptosis	(144)

GH, Growth hormone; IGF, Insulin-like factor; cTEC, Cortical Thymic Epithelial Cells; mTEC, Medullary Thymic Epithelial Cells; ETP, Early Thymic Progenitors; RTE, Recent Thymic Emigrants. Down arrow, decreased; Up arrow, increased.

Despite these important results, using GH as an anti-ageing drug is controversial since the treatment with the hormone may produce some undesirable side effects, including neoplasms, fluid retention, hyperinsulinemia, and insulin resistance (145, 146). Nevertheless, the recent pilot clinical trial by Fahy and colleagues seemed to find the solution to this problem, studying a cohort of healthy males aged 51 to 65 years, they administered GH (0.015 mg/kg) in conjunction with dehydroepiandrosterone (DHEA) (50 mg) and metformin (500 mg), to minimize the diabetogenic effect of GH. After one year of treatment, they found an increase in thymus size, high numbers of RTEs and naïve T lymphocytes, with simultaneous reduction of thymic fat content. The volunteers only exhibited mild side effects, illustrating that it is possible to achieve GH-based thymus rejuvenation without further damage to the aged organism (140).

Restoring effects like those described above for GH were observed in animals exposed to IGF-1. The treatment with this growth factor and bone marrow cells from young mice restored thymus cellularity in old recipients (141). In immune reconstitution studies, immunosuppressed mice treated with IGF-1 showed increased numbers of thymocytes (142). Moreover, it has been suggested that old animals with high circulating levels of IGF-I tend to have higher absolute peripheral blood CD4^+^ T-cell counts (147). In keeping with this finding, it has been shown that IGF-1 can induce *in vitro* proliferation of CD4^+^ thymocytes in a co-culture with thymic microenvironmental cells (148). The authors also observed that IGF-1 enhanced mRNA expression of the ThPOK encoding gene *Zbtb7b*, a transcriptional factor involved in the thymocyte commitment to the CD4^+^ T-cell lineage.

Ghrelin production and the expression of its receptor are also reduced in the mouse thymus since 6 months-old of age, especially in the medullary region of the thymic lobules (40). The significance of this phenomenon was demonstrated in the studies using knockout mice for ghrelin and the receptor GHS-R. These animals, when young, did not present any thymic alterations. However, aged mice showed a more important reduction in the thymus size and cellularity, an increase in thymic adiposity and a reduction in bone marrow-derived T-cell progenitors (40). The lack of ghrelin signaling reduced the number of both cortical and medullary TEC, as well as the gene expression of AIRE and IL-7 (39). In preclinical studies, when the peptide was administered for two weeks in 14 months-old mice, there was an increase in the thymic size and cellularity, including the numbers of cTEC and mTEC, as well as thymocyte progenitors and naïve lymphocytes in the periphery (40). Also, following ghrelin administration, aging mice displayed an increase in thymus weight and cellularity (143). Similarly, stress-mediated thymus involution was attenuated by ghrelin treatment, including thymocyte apoptosis (144). It is relevant to mention that these effects are independent of the secretion of GH from the pituitary and hepatic IGF-1, indicating that ghrelin has a local action in the thymus. These data show that ghrelin may be a valuable target to revert age-related thymic atrophy.

Overall, the findings described in this section show that GH and related peptides, especially ghrelin and IGF-1, are potential therapeutic alternatives to restore thymic function, not only during aging but also in acute stressful conditions that induce impairment of thymus and T lymphocyte functions. Aging is associated with a high mortality rate caused by chronic diseases and poor response to vaccination, features that are consequences of the failure of the immune system (immunosenescence) and thymus involution. In this perspective, strategies to improve thymic function may help to reduce the burden in health systems, whether public or private (149, 150). For instance, age-related thymic atrophy is believed to contribute to the severity of COVID-19 in old patients, indicating that a functional thymus is required to better cope with infection by SARS-CoV-2 (151, 152). Indeed, a clinical study showed a correlation between the presence of fat in the thymus and severe symptoms of the disease, such as high lung inflammation and lymphopenia (153). Another retrospective study revealed that the absence of the thymus due the atrophy was related to the severity of pneumonia in COVID-19 aging patients (154). It has been proposed that COVID mortality and morbidity might be related to the decline of GH levels during aging in humans, and highlighted the possible use of this molecule, as its secretagogues, in preventing or ameliorating COVID-19 symptoms (155). This hypothesis is reinforced by the fact that lower levels of GH and IGF-1 correlate with the presence of lung inflammation in COVID-19 patients (52).

## Concluding remarks

The data summarized in the present review provide clear evidence of an intricate physiological intrathymic circuitry involving GH, IGF-1, ghrelin, and somatostatin, including the relationship with thymus involution. It is important to emphasize that this circuitry can be affected by pathological situations, particularly those that occur with acute thymus atrophy.

**Figure 2:**
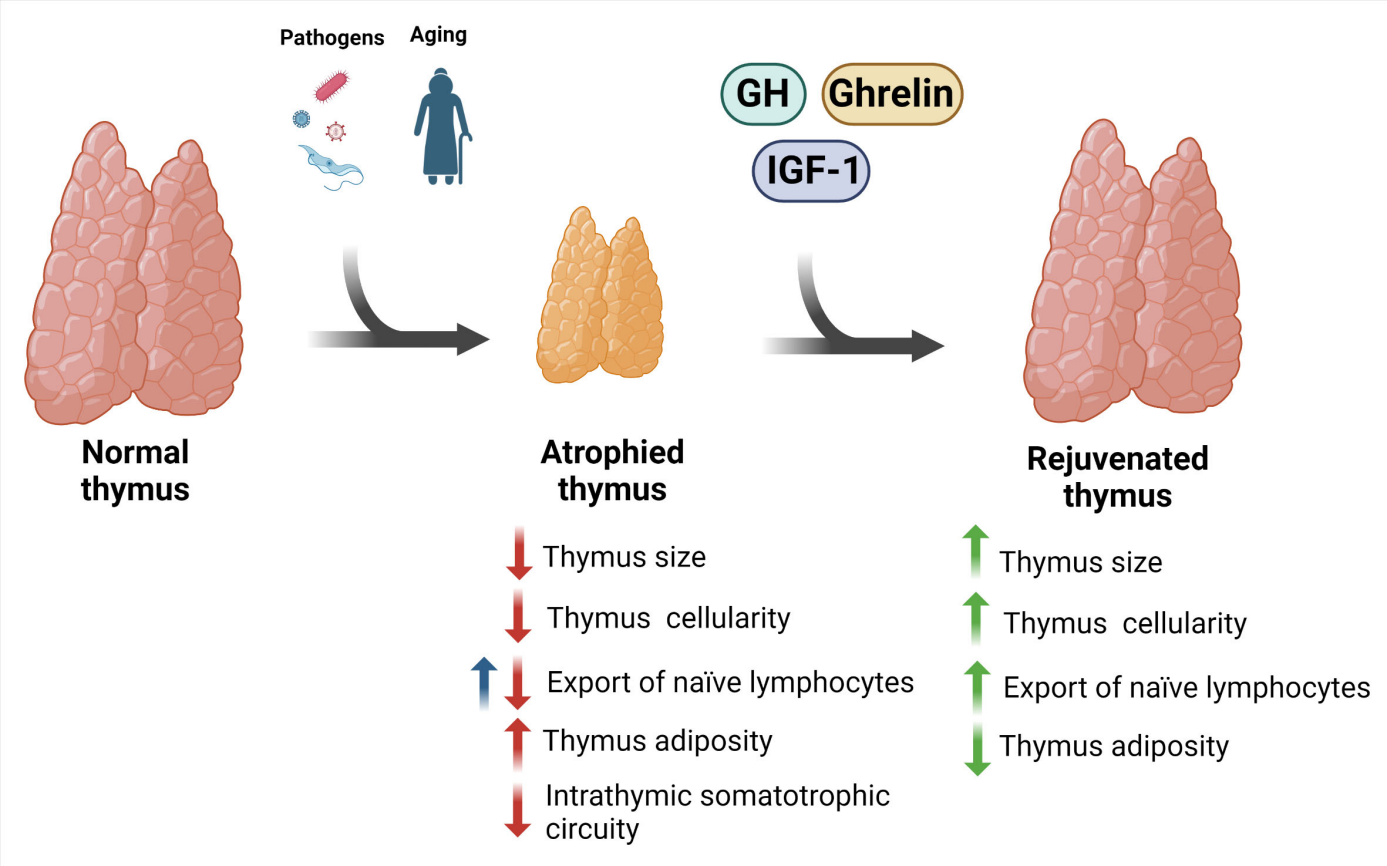
Potential rejuvenation of involuted thymus using GH and related peptides. Thymus involution is a physiological trait of aging that can also be triggered by pathogens. The consequence of this phenomenon is thymic atrophy, with impaired thymocyte maturation, increased cell death, increase in intrathymic adipocytes, and reduction in the export of naïve T lymphocyte to the periphery. Interestingly, there is evidence indicating an increase in the export of these cells in experimental Chagas disease (blue arrow). These changes were accompanied by a reduction in the GH, IGF-1, and ghrelin in the thymus. Importantly, some preclinical and clinical studies indicate that the treatment with these factors prevents and/or restore such atrophic thymus. Created with BioRender.com.

Overall, the findings place these molecules as potential therapeutic agents to reverse thymus involution, thus contributing to the replenishment of naive T lymphocytes in the periphery of the immune system. Similar reasoning can be made regarding the rejuvenation potential of the normal age-related atrophic thymus of elderly individuals, as schematically seen in **Figure 2**. It is worth mentioning that this proposed scheme needs further confirmatory studies, as most of the results are heavily based on preclinical studies, such as those on the effects of ghrelin on the aging thymus. Nevertheless, the results obtained in preclinical assays and the few data in humans tell us that the development of further clinical trials with representative cohorts and adequate controls may provide relevant information on the duration of the beneficial effects of these peptides upon the thymus, as well as on the possible side effects of their administration.

## Author contributions

WS conceived the idea for the review and defined the subtopics to be addressed. WS, MDSR, LPV, MPL, and FLP wrote the draft manuscript. DAM-d-C and WS reviewed the manuscript, and WS assisted in the submission process. All authors contributed to the article and approved the submitted version.
